# Adult Granulosa Cell Tumor in Pregnancy: A New Case and a Review of the Literature

**DOI:** 10.3390/healthcare9111455

**Published:** 2021-10-27

**Authors:** Sofia Guidi, Vincenzo Berghella, Giovanni Scambia, Anna Fagotti, Annalisa Vidiri, Stefano Restaino, Giuseppe Vizzielli, Frediano Inzani, Anna Franca Cavaliere

**Affiliations:** 1 Department of Obstetrics and Gynecology, Fondazione Policlinico Universitario Agostino Gemelli, IRCCS, 00168 Rome, Italy; giovanni.scambia@policlinicogemelli.it (G.S.); anna.fagotti@policlinicogemelli.it (A.F.); annalisavidiri@gmail.com (A.V.); giuseppevizzielli@yahoo.it (G.V.); 2 Division of Maternal-Fetal Medicine, Department of Obstetrics and Gynecology, Sidney Kimmel Medical College, Thomas Jefferson University, Philadelphia, PA 19107, USA; vincenzo.berghella@jefferson.edu; 3 Department of Obstetrics, Gyneacology and Pediatrics, Udine University Hospital, DAME, 33100 Udine, Italy; restaino.stefano@gmail.com; 4 Gynecopathology and Breast Pathology Unit, Department of Woman’s Health Science, Fondazione Policlinico Universitario Agostino Gemelli, IRCCS, 00168 Rome, Italy; frediano.inzani@policlinicogemelli.it; 5 Azienda USL Toscana Centro, Gynecology and Obstetrics Department, Santo Stefano Hospital, 59100 Prato, Italy; afcavaliere@hotmail.com

**Keywords:** granulosa cell tumor, pregnancy, recurrence, ovarian cancer, maternal outcome, fetal outcome, gynecology

## Abstract

Granulosa cell tumors are rare ovarian tumors that can arise during pregnancy. We present a new case of recurrent adult granulosa cell tumor (AGCT) in pregnancy and a systematic review of the literature. The new case described is a 41-year-old woman G5P1122 with a prior history of AGCT that was referred to our center at 29 weeks because of a symptomatic abdominal mass, compatible with a possible recurrence of AGCT. At 36 + 3 weeks, she underwent a cesarean delivery for preterm labor and a total hysterectomy with a radical surgical staging. A healthy female infant was delivered. The patient received a platinum-based chemotherapy, with a 26-month follow-up negative for recurrence. Analyzing our case with the four identified by the literature review, three were recurrent and two were primary AGCT. Only one required surgery for AGCT at 15 weeks, while another underwent chemotherapy in pregnancy. In the other three cases, surgery for AGCT was done at the time of cesarean delivery. There were three cases of preterm delivery. All five pregnancies resulted in the birth of live babies with weight adequate for gestational age. In conclusion, AGCT diagnosed in pregnancy is rare, reported in only five cases. All gave birth to live babies in the third trimester, and maternal outcome at up to 18 months showed no recurrence.

## 1. Introduction

Granulosa cell tumors account for 5–8% of all ovarian tumors [1]. They are classified according to histological and clinical presentation in two different types: Adult type and Juvenile type. The most common is the Adult type, which accounts for 95 % of all granulosa cell tumors, and it is usually diagnosed in the peri- and post-menopausal period. Menstrual irregularities, amenorrhea, and endometrial hyperplasia are common symptoms of Adult granulosa cell tumor (AGCT) [1]. The Juvenile type instead accounts for 5% of all granulosa cell tumors and is diagnosed in the first two decades of life. Granulosa cell tumors can also present during pregnancy, although this happens infrequently, in only 10% of cases [2]. The presentation of AGCT in childbearing age is unusual. We describe a new case of recurrent AGCT in pregnancy. In addition, we conducted a systematic review of the literature for primary or recurrent AGCT in pregnancy.

## 2. Materials and Methods

Data sources: a review of electronic databases (i.e., MEDLINE, Scopus, ClinicalTrials.gov, accessed on 20 October 2021, EMBASE, Science Direct, the Cochrane Library at CENTRAL Register of control trials, Scielo) was conducted from their inception until July 2021. Cases or case series of pregnant women with a history of AGCT diagnosed during pregnancy were identified by a review of the literature. A new case from our center was also identified. There were no restrictions of language and geographic location; articles in foreign languages were adequately translated by the authors. Demographic characteristics, details of the history of granulosa cell tumor, treatment and management, pregnancy and neonatology outcomes were reviewed in detail.

Search strategy and study selection: We followed the MOOSE guidelines to review the literature. The search terms used were “granulosa cell tumor” and “pregnancy”. Each article was assessed by three investigators S.G., V.B., and A.F.C. No contact with authors was necessary.

Inclusion criteria: The articles were included if the granulosa cell tumor was of the Adult type and if it was diagnosed or recurred during pregnancy.

Exclusion criteria: Articles on Juvenile granulosa cell tumor and articles not specifying the type of granulosa cell tumor were excluded. Tumors that were diagnosed after pregnancy were also excluded. Articles with multiple cases that had the variables analyzed as a group and did not give separate data for granulosa cell tumor type were excluded. Case reports of non-malignant lesions were excluded.

## 3. Results

Case report: A 41-year-old woman G5P1122 was referred to our center in April 2019 at 29 weeks of gestation because of a large symptomatic abdominal mass. The patient first identified a large bulging on the anterior abdomen in the supraumbilical region, which was later confirmed by her gynecologist. The ultrasound scan at the referring hospital showed a 9 cm mass, with hypo- and hyperechoic areas. Regarding her obstetrical history, the patient reported two spontaneous abortions, respectively in 2000 and 2011, an uncomplicated pregnancy and cesarean delivery at term in 2012, and a vaginal delivery at 26 weeks of gestation in 2018. During the current index pregnancy, no other maternal or fetal complications were diagnosed.

She had a history of AGCT, diagnosed in February 2011 with laparoscopic enucleation of a right ovarian cyst with intraoperative spillage. After histological evaluation, the definite diagnosis was AGCT. Both the mitotic index and the Ki67 proliferation index were low: two mitoses per 10 high power field (HPF) and 2%, respectively. Laparoscopic restaging three months later involved right salpingo-oophorectomy, lymph nodes sampling, and omental and peritoneal biopsies, which were all negative. Her AGCT was therefore FIGO stage Ic. No adjuvant treatment was prescribed, and the woman underwent regular oncological follow-up with negative results. Due to lack of recurrence, she was counseled that another pregnancy was not contraindicated.

**Figure 1:**
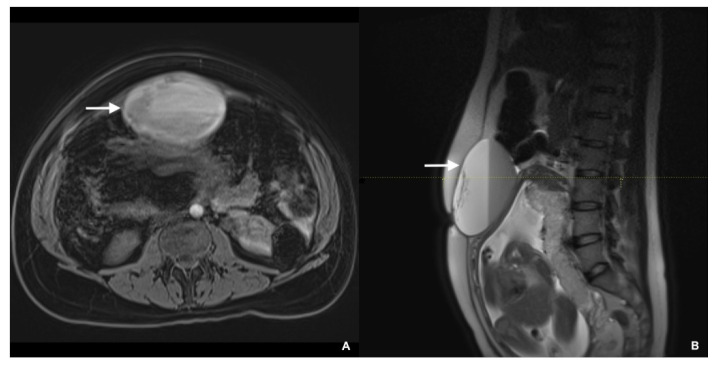
(**A**) Abdominal MRI axial T1-weighted spoiled gradient-echo post-Gd administration image showing a multiloculated anterior swelling of the uterine fundus, with a partially hematic liquid content. (**B**) Abdominal MRI sagittal T2-weighted image showing the mass extending cranio-caudally and the fetus in the gravid uterus.

Work-up of her mass during the index pregnancy at 29 weeks included magnetic resonance imaging (MRI) of the abdomen and pelvis, which revealed an anterior swelling of the uterine fundus measuring 9.6 × 6.7 × 12 cm^3^ extending cranio-caudally, with a partially hematic liquid content (Figure 1). Given the usual slow progression of AGCT and its prognosis, she was closely followed with ultrasonography at 2–3 weeks intervals. No changes were detected in morphology and dimensions of the mass, with no new findings.

Due to uterine contractions, repeat cesarean delivery originally planned for 37 weeks was performed at 36 + 3 weeks, under general anesthesia. A healthy female infant weighing 2570 g was delivered. The APGAR scores at 1 and 5 min were both 9. The multilocular solid cystic mass measuring about 10 cm localized at the uterine fundus was surgically removed. Masses smaller than 1 cm were observed in the left ovary (the right one was removed in 2011), bilaterally in the uterosacral ligaments, in the pouch of Douglas pouch and in the vesico-uterine fold. The frozen section of a sample performed on the largest mass revealed a solid proliferation of monomorphic epithelioid cells compatible with a recurrence of AGCT. Radical surgical staging was then performed at the time of cesarean with total hysterectomy, left salpingo-oophorectomy, excision of all macroscopically visible nodules in the peritoneum, and infracolic omentectomy. There were no pelvic or paraaortic bulky nodes, confirmed at intraoperative ultrasound.

**Figure 2:**
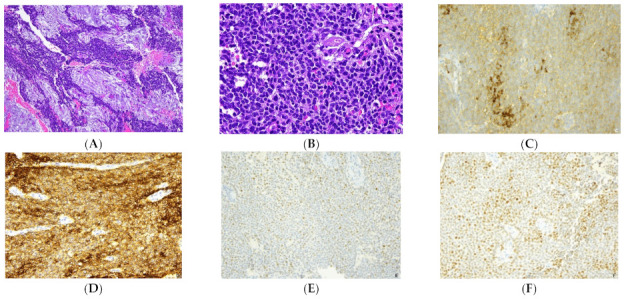
The histology of the AGCT shows a diffuse-trabecular pattern with areas of oedema and luteinisation (**A**) composed by monotonous small cells with scant cytoplasm and nuclei with occasional grooves (**B**). At immunohistochemistry neoplastic cells are positive for inhibin (**C**), CD56 (**D**) and they exhibit a significant expression of estrogen (**E**) and progesterone (**F**) receptors. H&E (**A**,**B**) immunoperoxidase (**C**–**F**).

The final pathological diagnosis confirmed the recurrence of the AGCT. The tissue was characterized by a proliferation of a solid-trabecular structure containing epithelial-like elements of medium size, relatively monomorphic with “nuclear grooves” (Figure 2). It showed a moderate mitotic activity, with 6 mitoses per 10 HPF. The Ki67 (MIB1 monoclonal, Dako, predilute) proliferation index was estimated to be 25%. The immunohistochemistry revealed positivity of neoplastic cells Inhibin-alpha (R1 monoclonal, Dako, predilute), CD56 (NCAM) (123c3 monoclonal, Dako, pedilute), S-100 (polyclonal, Dako, predilute), CD99 (12E7 monoclonal, Dako, predilute) and WT1(6F-H2 monoclonal, Dako, predilute); neoplastic cells showed negativity for EMA (E29 monoclonal, Dako, predilute), CK7 (OV-TL12/30 monoclonal, Dako, predilute) excluding the epithelial nature of the cells, negativity for synaptophysin (SP11 monoclonal, Roche, predilute) and chromogranin A (LK2H10 monoclonal, Roche, predilute) excluding neuroendocrine differentiation, and negativity for smooth muscle actin (HHF35 monoclonal, Dako, dilute 1:50) and desmin (D33 monoclonal, Dako, dilute 1:50) excluding the leiomuscolar nature. Estrogen receptors (ER) (SPI monoclonal, Roche, predilute) and profesterone receptors (PR) (1E2 monoclonal, Roche, predilute) were also performed in the option of a possible hormonal therapy and 15% of the neoplastic cells were weakly positive for ER and 75% of the neoplastic cells were positive for PR.

According to NCCN guidelines (ver. 2.2019), the patient was counseled for prolonged strict follow-up every 6 months for the first two years and then annually, versus adjuvant treatment, either with chemotherapy (CT) or hormone therapy. The patient elected CT treatment with paclitaxel and carboplatin, but after the first paclitaxel administration she experienced an allergic reaction. The patient underwent six cycles of CT with carboplatin every 21 days for 6 months. She has been counseled that AGCT generally has a favorable prognosis with an excellent estimated overall 5-years and 10-years survival of 97% and 95%, respectively [3]. The patient was also told of the possibility of a recurrence, as in patients with recurrent GCT, the disease-free interval can be lower [4]. The patient has been in follow-up for 26 months, and there has been no evidence of recurrence or metastasis.

**Figure 3:**
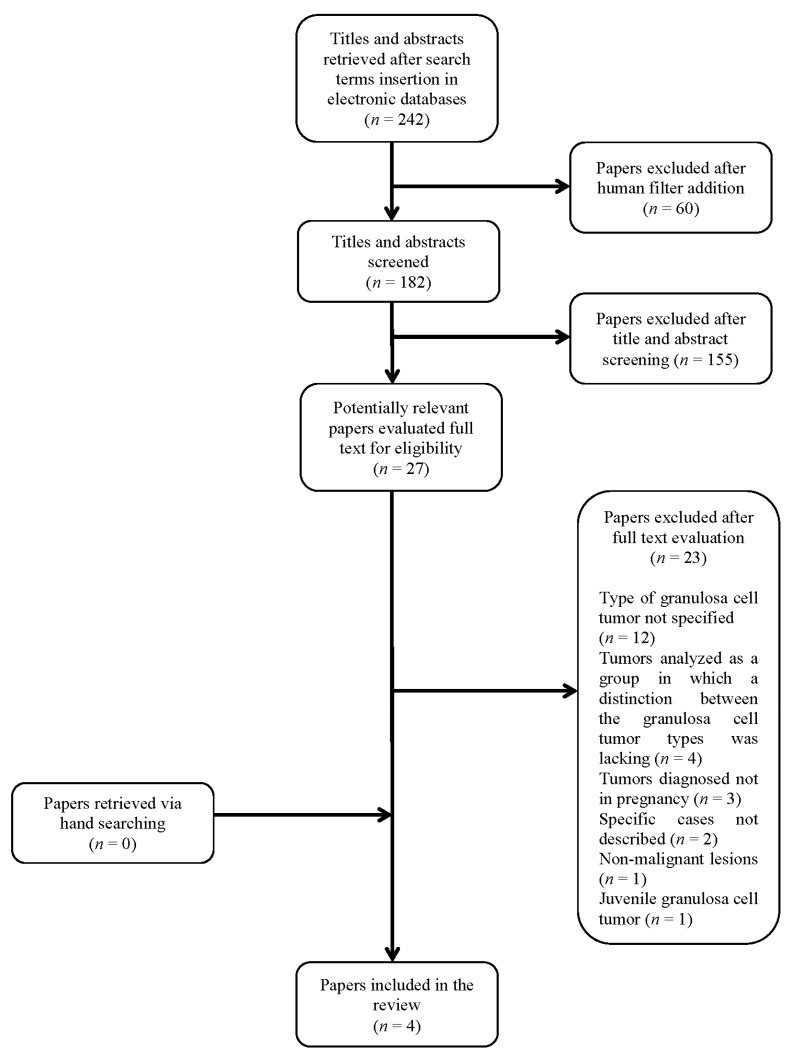
Cases of AGCT presenting or recurring in pregnancy were included, 4 from review of the literature [5,6,7,8] and our new case. Patients’ age ranged from 23 to 41. Three of the 4 cases reporting gravidity and parity were primigravid women. Three were primary, and 2 recurrent AGCT. In the 3 recurrent, previous surgery involved at least a salpingo-oophorectomy (Table 1).

Review of the literature: Our search yielded initially 242 results. After adding the filter “human” the number of publications decreased to 182. The references evaluation yielded no other pertinent case. The titles and abstracts were screened by investigators (S.G., V.B., A.F.C.), and 155 articles were excluded. The remaining 27 articles considered potentially relevant underwent a full text and references evaluation and 23 of them were excluded (Figure 3, Table 1).

Of the four cases with details available on symptoms, three had abdominal symptoms, while one was asymptomatic. Radiologic work-up for the abdominal symptoms always included an US, and sometimes also an MRI, in the second or early third trimester. Two cases were instead detected at cesarean delivery, at 32 weeks and at term. Tumor was localized in one of the ovaries in four out of five cases, and in the remaining case (ours), in the fundus of the uterus. Tumor dimensions were reported either by radiologic work-up or in surgery (Table 2).

Surgery during pregnancy was done in only one case, a laparoscopic left adnexectomy at 15 weeks [5]. In three other cases, surgery was done at the time of cesarean delivery, in the third trimester. These four cases did not have chemotherapy in pregnancy, while the fifth case [6] was given chemotherapy in pregnancy and also postpartum, and had surgery only 8 months postpartum (Table 3).

In terms of obstetrical outcome, three cases were delivered at term or near term [5,7], while two required delivery at 30 weeks for preterm labor [6] and 32 weeks for placental abruption [8]. The five live babies were of birthweight adequate for gestational age in the four that reported birthweight (Table 4).

Of the four cases that had surgery in pregnancy or at delivery but no chemotherapy, only one required postpartum surgery [8]; three also had chemotherapy (always including cisplatinum or carboplatinum), while in one postpartum, chemotherapy was not described (Table 5). Unfortunately, only short-term follow-up of the maternal AGCT, up to 18 months, was available in four cases. The postpartum follow-up showed no recurrence in all four cases.

## 4. Discussion

Our review of the literature revealed that AGCT diagnosed as either primary or recurrent tumor in pregnancy is rare, reported in only five cases. Surgery and chemotherapy during pregnancy was needed in only one case, while the other three had surgery at cesarean delivery. All gave birth to live babies in the third trimester, and maternal outcome at up to 18 months showed no recurrence. Several other cases of AGCT in pregnancy reported in the literature do not specify or distinguish type of granulosa cell tumor (Figure 3).

Granulosa cell tumors usually manifest as large unilateral masses with nonspecific symptoms and specific diagnostic criteria. The recommended management is surgical, also necessary for tumor staging [1]. These tumors are characterized by a late recurrence, so long follow-up is advised. If surgical removal of only one ovary is necessary, women with a history of AGCT can have a spontaneous pregnancy.

The strength of our study is that to our knowledge there are no other reviews of AGCT occurring as primary or recurrent tumor during pregnancy. Non-English language studies were not excluded. Limitations were inherent to the details provided by the reports identified, many reports did not specify or distinguish the type of GCT. The cases included not always provided all details, and postpartum follow-up was short, at maximum only 18 months.

## 5. Conclusions

As AGCT is often initially managed with unilateral oophorectomy in women of reproductive age, pregnancy in women with this history is possible. AGCT in pregnancy has been reported in detail only in five cases. Surgery and chemotherapy are seldom necessary during pregnancy, as this is usually a slow growing tumor. Short-term maternal and perinatal outcomes are in general favorable, with surgery for AGCT often done at cesarean, live births, and postpartum platinum chemotherapy. Care with a multidisciplinary team including gynecologic oncology surgeons, radiologists, obstetricians and maternal-fetal specialists, pathologists, anesthesiologists and neonatologists is recommended. Given the presence of only five total cases of AGCT in pregnancy in the literature, more research is needed.

## Figures and Tables

**Table 1:** Patient characteristics and adult granulosa cell tumor history.

First Author	Year	Patient Age	Gravidity and Parity	Primary/Recurrent	Previous Oncological Surgeries before Pregnancy
Guidi	2019	41	G5P1122	Recurrent	Laparoscopic enucleation of an ovarian cyst on the right ovary initially, then right salpingo-oophorectomy, lymph nodes sampling, and omental and peritoneal biopsies
Aymen	2016	30	G1P0	Primary	NS
Roy	2014	23	G1P0	Primary	NS
Agarwal	2011	26	G1P0	Recurrent	Right salpingo-oophorectomy
Fernandez-cid	2011	35	NS	Primary	NS

NS, not stated.

**Table 2:** Adult granulosa cell tumor symptoms, work-up and specifics in pregnancy.

First Author	Symptoms/Signs in the Index Pregnancy Which Prompted Work-Up	Week at Radiologic Work-Up or Intraoperative Diagnosis	Radiologic Work Up	Tumor Localization	Tumor Dimension
Guidi	Bulging of a symptomatic abdominal mass, that was palpable and visible	29	US and MRI	Uterine fundus	9.6 × 6.7 × 12 cm^3^ (MRI)
Aymen	Intermittent abdominal unspecific pain	32 (CD)	NS	Right ovary	40 × 30 cm (intraoperative)
Roy	NS	At term (CD)	NS	Right ovary	10 × 10 × 5 cm (intraoperative)
Agarwal	Acute pain and progressive abdominal distension	20	US and MRI	Left ovary	9.7 × 7.7 cm (US-MRI)
Fernandez-cid	Asymptomatic; tumor detected at routine abdominal ultrasound	NS	US	Left ovary	17 × 14.1 × 11.8 cm (US)

CD, tumor detected at cesarean delivery. NS, not stated.

**Table 3:** Tumor management during pregnancy.

First Author	Week at Surgery	Surgery in Pregnancy	Surgery at Delivery	Intraoperative Findings and Sites Positive for AGCT	Chemotherapy during Pregnancy
Guidi	36 + 3	No	Total hysterectomy, left salpingo-oophorectomy, excision of every macroscopically visible nodules in the peritoneum, and infracolic omentectomy	Left ovary, bilaterally in uterosacral ligaments, vesico-uterine fold and douglas pouch.	No
Aymen	32	No	Right adnexectomy and partial omental removing	Omentum	No
Roy	At term	No	Right sided ovariectomy	NS	NS
Agarwal	Not applicable	No	No	Significant ascites, peritoneal implants, omental thickening, enlarged paraaortic lymph nodes and bilateral pleural effusion.	Adriamycin–Vincristine (week 21)
Fernandez-cid	15	Laparoscopic left adnexectomy	No	NS	NS

NS not stated.

**Table 4:** Obstetrical outcomes.

First Author	Gestational Age Delivery (Weeks + Days)	Mode of Delivery	Pregnancy Outcome	Birth Weight (Grams)	Apgars at 1 and 5 min
Guidi	36 + 3	Planned cesarean done early for preterm labor	Live birth, female	2750	9/9
Aymen	32	Emergency cesarean for placental abruption	Live birth	1925	2/5 *
Roy	At term	Emergency cesarean for labor obstruction	Live birth, male	2500	NS
Agarwal	30	Vaginal after preterm labor	Live birth, male	1200	NS
Fernandez-cid	39	Planned cesarean	Live birth, female	NS	NS

* 10 at 10 min, NS not stated.

**Table 5:** Post-delivery tumor management and outcome.

First Author	Surgery after Pregnancy	Chemotherapy after Pregnancy	Maternal Oncologic Outcome
Guidi	No	Six cycles of Carboplatin	Follow-up at 26 months negative for recurrence
Aymen	Total hysterectomy, left adnexectomy, total omentectomy, appendicectomy, and multiple peritoneal biopsies	Four cycles of BEP protocol (after surgery in pregnancy)	Follow-up at 18-months negative for recurrence
Roy	NS	Yes (type not described)	NS
Agarwal	Total abdominal hysterectomy and left salpingo-oophorectomy (After the chemotherapy and 8 months postpartum)	Six cycles of cisplatin regimen	Follow-up at 10 months after delivery negative for recurrence
Fernandez-cid	No	NS	Asymptomatic and at last US no recurrences

BEP, bleomycin, etoposide, and cisplatinum; CT, chemotherapy, NS, not stated.

## Data Availability

The data presented in this study are available on request from the corresponding author.
